# Comparison between a novel helical and a posterior ankle–foot orthosis on gait in people with unilateral foot drop: a randomised crossover trial

**DOI:** 10.1186/s12984-023-01184-x

**Published:** 2023-05-11

**Authors:** David Gasq, Raphaël Dumas, Benoit Caussé, Marino Scandella, Pascal Cintas, Blandine Acket, Marie Christine Arné-Bes

**Affiliations:** 1 Service des Explorations Fonctionnelles Physiologiques, CHU de Toulouse Rangueil, 31059 Toulouse, France; 2 Toulouse NeuroImaging Center, Université de Toulouse, Inserm (UMR 1214), UPS, 31024 Toulouse, France; 3 Univ Lyon, Univ Gustave Eiffel, Univ Claude Bernard Lyon 1, LBMC UMR_T 9406, 69622 Lyon, France; 4 Centre d’évaluation et de traitement de la douleur, service de neurochirurgie, CHU de Toulouse Purpan, 31059 Toulouse, France; 5 Centre de référence des maladies neuromusculaires, département de Neurologie, CHU de Toulouse Purpan, 31059 Toulouse, France; 6 Laboratoire d’analyse de la marche, Hôpital des Enfants, CHU de Toulouse Purpan, 31059 Toulouse, France

**Keywords:** Orthotic devices, Foot drop, Biomechanics, Six-minute walk test, Neuromuscular disease, Peripheral neuropathy

## Abstract

**Background:**

Neuromuscular disease and peripheral neuropathy may cause drop foot with or without evertor weakness. We developed a helical-shaped, non-articulated ankle–foot orthosis (AFO) to provide medial–lateral stability while allowing mobility, to improve gait capacity. Our aim was to evaluate the effect of the helical AFO (hAFO) on functional gait capacity (6-min walk test) in people with peripheral neuropathy or neuromuscular disease (NMD) causing unilateral drop foot and compare with a posterior leaf spring AFO (plsAFO). Secondary aims were to compare functional mobility, 3D kinematic and kinetic gait variables and satisfaction between the AFOs.

**Methods:**

Single centre, randomised crossover trial from January to July 2017 in 20 individuals (14 with peripheral neuropathy and 6 with NMD, 12 females, mean age 55.6 years, SD 15.3); 10 wore the hAFO for the first week and 10 wore the plsAFO before switching for the second week. The 6-min walk test (6MWT), Timed Up and Go (TUG) test and 3D gait analysis were evaluated with the hAFO, the plsAFO and shoes only (noAFO) at inclusion and 1 week after wearing each orthosis. Satisfaction was evaluated with the Quebec user evaluation of satisfaction with assistive technology (QUEST).

**Results:**

Median [interquartile range] 6MWT distance was greater with the hAFO (444 m [79]) than the plsAFO (389 m [135], P < 0.001, Hedge’s g = 0.6) and noAFO (337 m [91], P < 0.001, g = 0.88). TUG time was shorter with the hAFO (8.1 s [2.8]) than the plsAFO (9.5 s [2.6], P < 0.001, g = − 0.5) and noAFO (10.0 s [2.6]), P < 0.001, g = − 0.6). The plsAFO limited plantarflexion during the loading response (plsAFO − 7.5 deg [6.0] vs. noAFO -13.0 deg [10.0], P = 0.0007, g = − 1.0) but the hAFO did not (− 11.0 deg [5.1] vs. noAFO, P = 0.05, g = − 0.5). Quasi-stiffness was lower for the hAFO than plsAFO (P = 0.009, g = − 0.7). The dimensionless eversion moment was higher (though not significantly) with the hAFO than noAFO. Neither orthosis reduced ankle power (P = 0.34). Median total QUEST score was higher for the hAFO (4.7 [0.7]) than the plsAFO (3.6 [0.8]) (P < 0.001, g = 1.9).

**Conclusions:**

The helical orthosis significantly and considerably improved functional gait performance, did not limit ankle mobility, increased lateral stability, though not significantly, and was associated with greater patient satisfaction than the posterior leaf spring orthosis.

*Trial registration* The trial began before registration was mandatory

## Introduction

Diseases that affect the strength of the dorsiflexor muscles, such as stroke, peripheral neuropathy and neuromuscular disease (NMD), can result in foot drop. Foot drop restricts gait capacity and induces compensatory strategies, such as increased hip and knee flexion, to clear the foot and avoid tripping [1, 2]. Foot drop may be associated with evertor weakness, which reduces medial–lateral stability and may also only become apparent as the muscles fatigue.

Ankle–foot orthoses (AFOs) are commonly prescribed, conservative methods to reduce foot drop and improve gait capacity [3]. They are designed to compensate for the lack of active dorsiflexion by preventing plantar flexion during the swing phase of gait [3]. Although AFOs do not normalise the gait pattern, they reduce kinematic and kinetic anomalies [4, 5], the aim of which is to reduce compensatory strategies and risk of falls, and increase gait capacity, which may in turn facilitate participation [6]. The use of AFOs to improve gait capacity has been well studied in patients with stroke, however their use in people with NMD or peripheral neuropathy has been little evaluated.

Many types of AFOs have been designed and different materials have been used [7, 8], however, in France, posterior leaf spring AFOs made of thermoformable polypropylene are frequently used because they are reimbursed [9]. These orthoses effectively prevent foot drop and are compact and low cost, however they are not designed to provide stability in the frontal plane (they must be worn with high shoes), are not lightweight and are unable to restore energy to aid propulsion.

According to Alam et al. (2014), the ideal AFO must meet the following design specifications: be lightweight, compact, efficient, and untethered, prevent drop foot during swing while allowing normal ankle motion during other phases, and assist push off if necessary [8]. People with NMD or peripheral neuropathy often have associated weakness of the ankle evertor muscles, resulting in an unstable base of support during stance, therefore AFOs must also provide medial–lateral stability [10]. However, AFOs can only effectively improve gait capacity if they are actually worn [11–13]. Comfort and aesthetics are important criteria which contribute to compliance with AFOs [14] and must therefore be considered in the design.

Few commercialised orthoses currently meet all these criteria: carbon orthoses are lightweight and compact and may provide some push off assistance [15, 16], however, in our clinical practice we have found that if they provide lateral stability, this is often at the expense of mobility in the sagittal plane. Several attempts have been made to produce AFOs that specifically provide lateral stability. For example, Bishop et al. 2009 used an interesting design to prevent inversion during walking and running, however the AFO held the foot in maximal dorsiflexion, therefore altering the normal gait pattern [17]. Another group designed an ankle control strap to prevent unwanted eversion during gait [18], however it was worn on a bulky, inaesthetic AFO. Furthermore, AFOs may reduce propulsion forces, even in people who have sufficient plantarflexor strength to generate such forces [4, 16]. Since people with NMD often have plantarflexor weakness, a further requirement of an AFO is therefore to restore energy to assist push-off, or at least not reduce propulsive forces. Spiral orthoses have been proposed in the past to overcome these issues [19]. They are lightweight and can control motion in all planes. However, 3D gait kinematics have not been compared between spiral orthoses and AFOs that are typically prescribed today.

One of the authors of the present study (BC) designed and developed a new type of carbon fibre orthosis with a helical shape that would: (1) prevent foot drop in swing, (2) allow normal ankle range of motion, (3) not reduce propulsion and (4) provide medial–lateral stability of the ankle. It was also intended to be lightweight, and aesthetically acceptable. Such an orthosis could improve gait capacity.

The purpose of this study was first to determine if the orthosis improved gait capacity, which is the main aim of the wearer (activities level of the International Classification of Functioning, Disability and Health [6]), and second to determine if improvements in gait capacity with the orthosis could be related to improvements in the 4 criteria described above (body function and structures level of the International Classification of Functioning, Disability and Health).

The primary aim of this study was to evaluate the effect of this helical AFO (hAFO) on functional gait capacity (distance) in comparison with the effects of a posterior leaf spring AFO (plsAFO) or no AFO in patients with peripheral neuropathy or NMD causing drop foot with or without a concomitant loss of evertor strength. The secondary aims were to evaluate and compare functional mobility, 3D kinematic and kinetic gait variables and satisfaction between the two types of AFO.

## Methods

### Study design

We conducted a single centre, randomized crossover trial from January to July 2017. Two types of orthoses were evaluated: a posterior leaf spring AFO (plsAFO) and the specifically designed, helical AFO (hAFO). Participants were randomised to wear one of the orthoses in their usual environment for 1 week and the other orthosis for the following week. Randomisation of the order of the orthoses (plsAFO first or hAFO first) was performed using a computer-generated list created by the study clinical research organisation (4-block randomisation). Two doctors enrolled consecutive participants and performed the group allocation. The evaluations were performed in the same order. Neither the participants nor the evaluators were blinded to the condition.

**Fig. 1:**
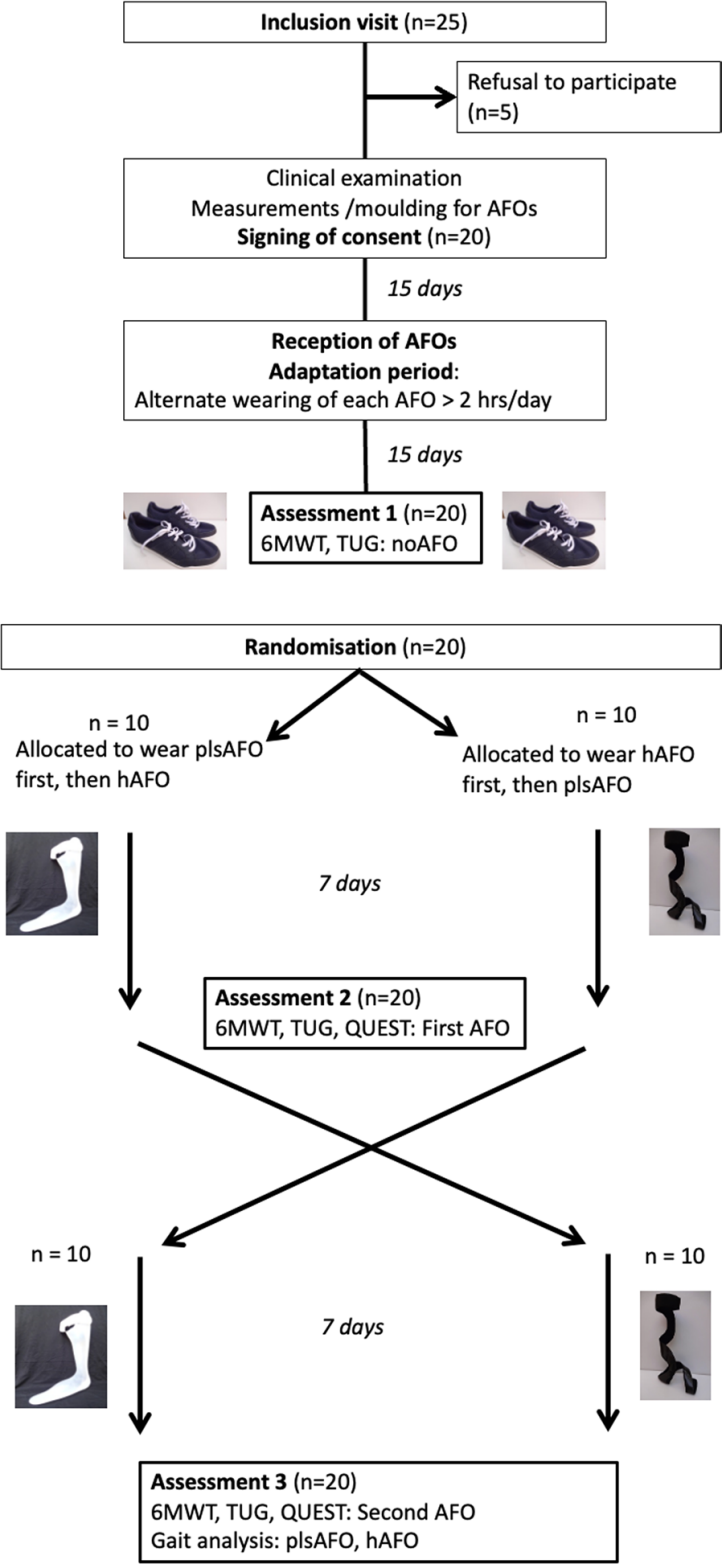
Flow chart of study schedule

The study schedule is shown in Fig. 1. The reporting of the study followed the 2010 CONSORT guidelines: extension for randomised crossover trials.

### Participants

According to the sample size calculation performed with G*Power 3.1 software (data from Nolan et al. 2009 [20]), power 90%, alpha risk 5%, effect size *f* 0.35), 20 participants were required.

Inclusion criteria were: adults (over 18 years old) with unilateral drop foot caused by peripheral neuropathy or NMD who usually wore an AFO, Medical Research Council (MRC) score of the dorsiflexor or evertor muscles < 3 or score of 3 on testing but the person complained of fatigue that resulted in foot drop during gait, MRC score of at least 4 in the hip and knee flexors and extensors and plantarflexors of the contralateral limb, able to walk safely without an AFO for at least 6 min (with or without gait aids), and able to understand simple instructions. Individuals were excluded if they had any lower limb spasticity, required bilateral assistive devices for gait, had undergone lower limb surgery in the 6 previous months that could affect gait, or if they were unable to provide written informed consent. Eligible individuals were consecutively screened during routine consultations in the Adult Neuromuscular Disease Department of Toulouse University Hospital. Those who agreed to participate (20 out of 25 individuals) attended the inclusion visit and signed the consent form.

### Ankle foot orthoses

**Fig. 2:**
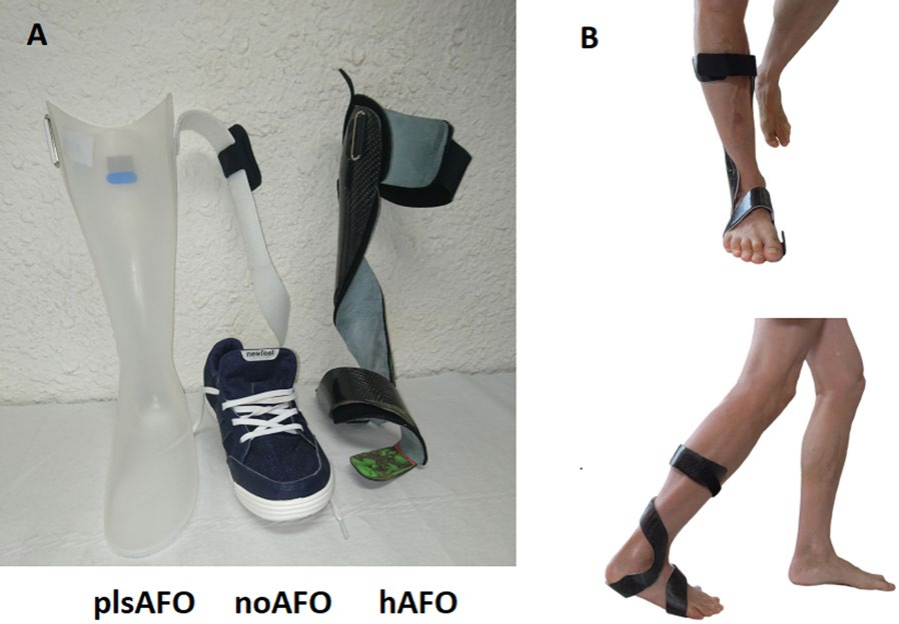
**A** Images of the posterior ankle foot orthosis (plsAFO), shoe worn by all participants (noAFO) and helical ankle foot orthosis (hAFO). **B** Frontal and lateral view of the hAFO

Participants underwent moulding for the fabrication of the plsAFO and the hAFO during the same visit. The orthoses were fabricated by a single orthotist (OCTO31, Colomiers, France) with 40 years of experience and provided to the participants 2 weeks later. The orthotist was independent, was not involved in the design and development of the hAFO, was not employed by InnovPulse (AFO manufacturer) and is not one of the authors. The ankle–foot orthoses are shown in Fig. 2.

Two ankle foot casts were made, one for each orthosis. For both, the ankle was positioned at 90° and the foot positioned so that the 2nd toe, considered to indicate the axis of the foot, was in line with the patella. Deformities in the frontal plane were not corrected. The shape of the orthosis (hAFO or plsAFO) was traced on the plaster positive.

After receipt of both AFOs, and prior to randomisation, participants were instructed to wear each one for at least 2 h per day (they could wear it all day if they wanted to), on alternate days (ie, the plsAFO one day and the hAFO the other day) during a two-week adaptation period (see Fig. 1). The purpose of this was to ensure the participants had experience of both orthoses. After randomisation, they wore one AFO for 7 days followed by the other for 7 days while performing their activities of daily living, according to the order determined by the randomisation.

#### Posterior leaf spring AFO

The plsAFO was made from a single piece of thermoformable polypropylene that encompassed the dorsal part of the leg and the sole of the foot (Fig. 2). A sheet of polypropylene (3–4 mm) was vacuum thermoformed over the plaster positive. Once cool, the trimline was traced and cut using an oscillating saw. The edges were rounded with a rotary power tool. No foam was used. A strap was fixed to the proximal part.

The same trim line was used for all plsAFOs. The proximal edge was 4 cm below the popliteal fossa. The anterior edge (medial and lateral) followed a line that passed from proximal to distal, just in front of the centre of the calf in the sagittal plane to the posterior border of the malleolus. The orthosis supported the medial arch of the foot to the head of the first metatarsal before flattening.

This orthosis prevented plantarflexion during swing phase and allowed passive ankle dorsiflexion during stance phase. It is commonly used in France [9] so was considered as an appropriate reference for comparison of the new helical orthosis. Although each plsAFO was made for each participant, we did not attempt to customise it to the participant’s impairments, e.g., to provide more lateral stability, because we wanted a homogenous comparator orthosis.

#### Helical AFO

The helical ankle–foot orthosis (hAFO) from InnovPulse (Vernaison, France) [21] [Patent no. EP2931189 (B1)] was a lightweight, non-articulated, carbon orthosis.

All the hAFOs were custom designed and fabricated from multiple layers of carbon. Layers of dry carbon fibre were superimposed between 2 closed end PVA bags over the plaster positive and epoxy resin was injected to bind the layers. The pieces were positioned over the tracing of the AFO on the plaster positive. The resin was then polymerised. The hAFO was cut along the trace using a diamond Dremel wheel. The edges were rounded on a rotary power tool before hand-finishing with fine-grain sandpaper. After cleaning with alcohol, EVA foam (1.5 mm) was fitted on the distal part of the hAFO, up to the lower edge of the lateral malleolus. The strap was then added on the proximal part of the orthosis.

The same trim line was used for all hAFOs: a helical shaped band passed laterally under the metatarsal heads, medially over the dorsum of the foot, under the heel, anterior to the lateral malleolus, behind the calf and fixed with the strap just distal to the knee (Fig. 2). The band formed a cup under the heel that provided medial–lateral support, to increase ankle stability in the frontal plane, but no posterior support, to allow full ankle mobility in the sagittal plane. It also allowed the normal degrees of freedom between the rear- and fore-foot.

### Evaluations

All participants attended 3 evaluation visits (Fig. 1) that were conducted by the same experienced rehabilitation physician.

Visit 1: the physician verified the inclusion criteria and collected demographic data (age, sex and body mass index). Strength of the dorsiflexor, evertor and plantar flexor muscles was evaluated with the MRC to characterise the participants [12]. The six-minute walk test (6MWT) and timed up and go test (TUG) were performed in the noAFO condition (shoes only).

Visit 2: one week after wearing the first orthosis, the 6MWT and TUG test were performed with the participant wearing the first orthosis, and the Quebec user evaluation of satisfaction with assistive technology (QUEST) was rated. A 10-min rest was allowed between each test. Participants all wore the same type of shoes (ordinary, low trainers, Fig. 2), which were provided for the evaluations.

Visit 3: 1 week after wearing the second orthosis, the same tests were performed in the same conditions as for Visit 2 with the participant wearing the second orthosis. In addition, 3D gait analysis was performed in all 3 conditions.

### Outcome measures

#### Primary outcome

##### 6-min walk test (6MWT)

The 6MWT is a functional measure of gait capacity that evaluates the distance walked over a period of 6 min. The test was performed in a 30-m straight corridor. Participants were instructed to walk as far as possible within the 6 min and standardised instructions were used [22]. Walking aids such as sticks were permitted. One research assistant recorded the total distance walked for each participant. One trial was performed. The MCID for the 6MWT has been estimated to be around 34 m in patients with facioscapulohumeral muscular dystrophy (FSH) [23] and a study in people with stroke reported a change of 50 m to indicate a substantial meaningful change [24].

#### Secondary outcomes

##### Timed up-and-go test (TUG)

The timed up and go (TUG) test measures mobility, including static and dynamic balance [25, 26]. The time (in seconds) taken for a participant to stand up from a chair with a back rest and arm rests, walk three meters, turn around, return to the chair, and sit down again was recorded. Walking aids such as sticks were permitted. Two trials were performed, and the best result was used in the analysis. The MCID for the TUG test varies considerably in the literature, from 1 s for patients with hip osteoarthritis [27] to 3.4 s in patients with lumbar degenerative disc disease undergoing surgery [28].

##### The Quebec user evaluation of satisfaction with assistive technology (QUEST)

The QUEST 2.0 (validated in French version) was used to evaluate participants’ level of satisfaction with each AFO [29]. The questionnaire comprises 12 satisfaction items; 8 questions relate to the device features: dimensions, weight, ease of adjustment, safety and security, durability, ease of use, comfort, and effectiveness; and 4 questions relate to services provision: delivery, repairs and maintenance, professional services and follow up services. Responses are rated on a 5-point scale ranging from: 1: “not satisfied at all”; to 5: “very satisfied”.

##### Harms

Harms were collected at each visit: participants were asked if they had experienced any skin lesions or falls or any other problems they believed were related to the AFO.

##### Adherence

Participants reported wearing both AFOs as requested, i.e., during activities of daily living.

##### 3D gait analysis

Kinematic and kinetic gait variables were recorded on a 10 m runway with 8 cameras (Vicon Oxford Metrics, recording frequency 100 Hz) and 2 force platforms (AMTI OR6-7, Advanced Mechanical Technology Inc., Watertown, MA, recording frequency 1000). A set of 16 markers was fixed to bony landmarks according to the Helen Hays model [30]. Ten trials were recorded (following at least 3 familiarisation walks over the runway) for each condition.

Gait speed was fixed at the individual’s speed measured during the 6MWT in the noAFO condition (mean 0.95 m/s, SD 0.25) so that changes in gait speed between conditions would not confound kinematic and kinetic results. A running light-emitting diode by the side of the walkway set to the participant’s gait speed was used to guide them.

Trials in which participants placed the evaluated foot correctly on the force platforms were considered and were included in the analysis if at least 4 gait cycles with complete kinematic and kinetic data were available for the participant. Standardised kinematic [31] and kinetic data [32] were processed with a custom Matlab toolbox [33]. Three-dimensional ankle joint angles and inter-segmental moments, powers, work, and quasi-stiffness (computed as the slopes of the best-line fits of the linear parts of the moment–angle curve) [34] were calculated [35]. All kinematic and kinetic data (except angles) were made dimensionless [36] to reduce inter-participant variability. The variables analysed were peak dorsiflexion in early stance, peak plantarflexion in mid stance, peak dorsiflexion in swing, maximum dorsi/plantar flexion during swing, peak extension moment, peak inversion moment, peak eversion moment, peak negative power, peak positive power, negative work value, positive work value, quasi-stiffness value for the first linear part of the plantar/dorsiflexion moment–angle curve, quasi-stiffness value for the second linear part of the plantar/dorsiflexion moment–angle curve.

The eversion moment of the ankle joint was considered relevant to indicate ankle stability.

### Statistical analyses

The data did not follow a normal distribution (Shapiro–Wilk test and visual inspection). The Friedman test was used to compare the effects of the conditions (hAFO, plsAFO and noAFO) on the 6MWT, TUG test and the 13 variables from the 3D gait analysis. When significant differences were found, the Wilcoxon test was used post-hoc to determine which conditions differed. The Wilcoxon test was also used to compare the hAFO and plsAFO conditions on the two QUEST subscales (device and services) and the total QUEST score. To control the false discovery rate, a Bonferroni procedure was performed to adjust the alpha risk threshold (P-value) at 0.0031 (0.05/16) for the 13 Friedman tests and 3 Wilcoxon tests (n = 3) and at 0.017 for the 3 post-hoc Wilcoxon tests (n = 3) for each significant Friedman test. Effect sizes were also calculated using Hedges’ g adapted for dependent data [37] since they are less dependent on sample size and variability of data [38, 39]. Effect sizes were classed as small (0.2), moderate (0.2–0.8) or large (> 0.8) [37]. A difference was considered statistically significant if the P-value was below the adjusted P-value and the Hedges’ g confidence interval did not include zero. Median values and interquartile ranges (IQR) are presented. Matlab software was used for the statistical analyses.

## Results

Twenty individuals (8 men, 12 women, median age 53.4 years, IQR 23.3, range 29.5–79.4) were included: n = 14 with peripheral neuropathy (n = 11 with muscle weakness due to sciatic nerve injury, n = 1 anterior acute poliomyelitis, and n = 2 with Charcot-Marie-Tooth disease type Ia) and n = 6 with FSH. Median dorsiflexor strength was 3.0 (IQR 1.0, range 1–4), median eversion strength 3.0 (IQR 0.3, range 2–4) and median plantarflexor strength was 4.5 (IQR 1.0, range 3–5). All analyses were conducted by original assigned groups. Details of the baseline assessment are provided in Table 1.

**Table 1:** Demographic characteristics and baseline evaluation data according to randomisation group

	Group randomised to wear hAFO then plsAFO	Group randomised to wear plsAFO then hAFO
Age (years)	67.5 (18.2)	47.4 (13.5)
Males/Females (n)	5/5	3/7
Peripheral neuropathy / NMD (n)	8/2	6/4
Time since onset (months)	66.5 (140.5)	60 (116.8)
Dorsiflexor strength	3.0 (1.0)	2.5 (1.8)
Evertor strength	3.0 (0)	3.5 (1.0)
Plantarflexor strength	4.0 (1)	5 (1.8)
6MWT (noAFO)	355.5 (110.8)	321.0 (89.5)
TUG (noAFO)	10.0 (1.6)	9.6 (4.2)

Data are median (interquartile range)

*hAFO* helical ankle foot orthosis, *plsAFO* posterior ankle foot orthosis, *6MWT* six-minute walk test, *TUG* timed up and go test, *SD* standard deviation, *IQR* interquartile range, *n* number

### 6MWT

**Fig. 3:**
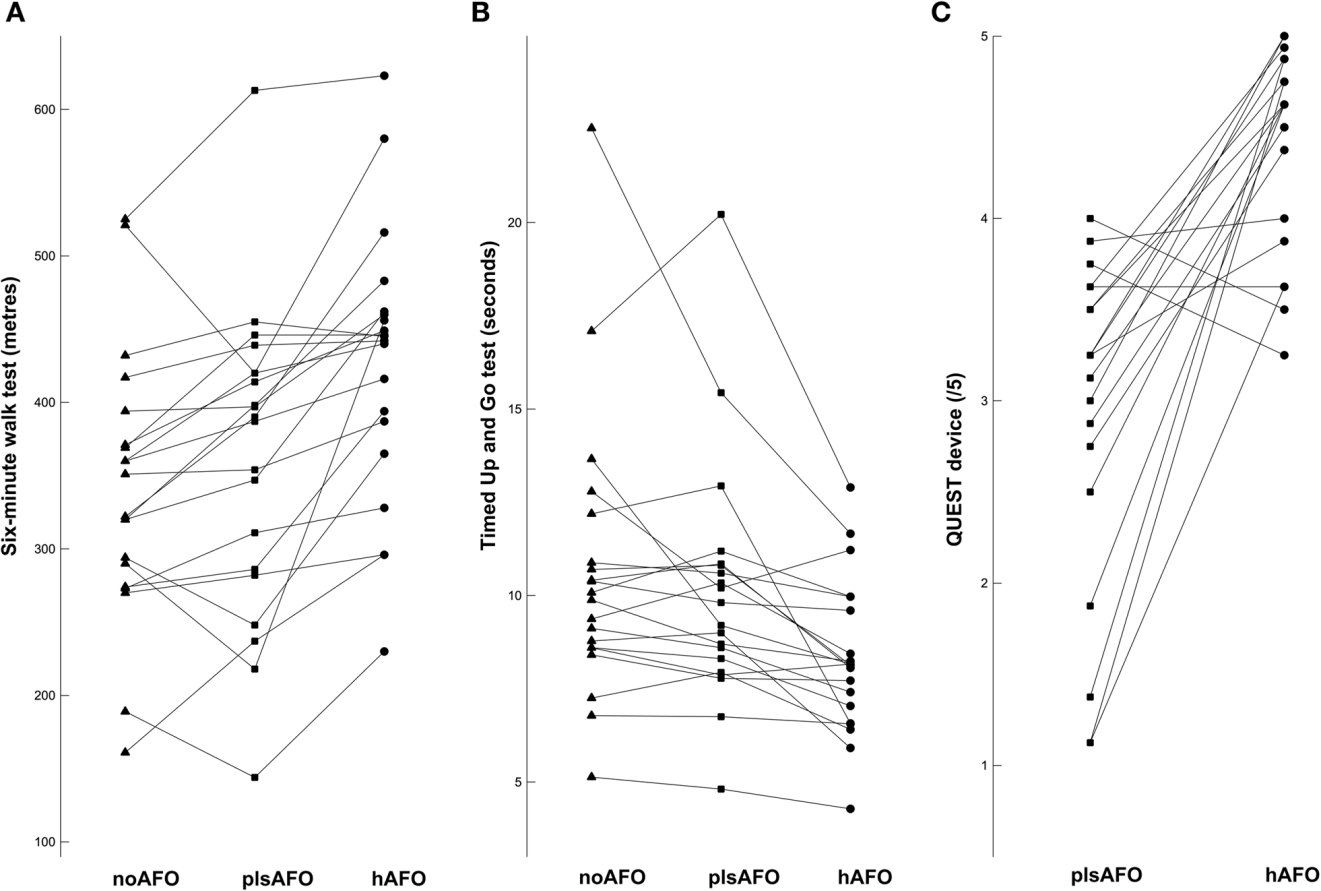
Results for each participant for A: the six-minute walk test, B: the timed up and go test for the three conditions (noAFO: shoes only, plsAFO: posterior ankle foot orthosis; hAFO: helical ankle foot orthosis) and **C** the the device sub-section of the Quebec evaluation of satisfaction

All 20 participants completed the 6MWT. 6MWT distance was significantly affected by the condition. The post-hoc analysis showed that this distance was significantly greater with the hAFO than the plsAFO (Table 2). Data for each participant are shown in Fig. 3A.

**Table 2:** Results of the 6MWT, TUG and 3D gait analysis and comparisons for each condition

	hAFO	plsAFO	noAFO	Friedman test	hAFO vs. plsAFO	hAFO vs. noAFO	plsAFO vs. noAFO
	Median (IQR)	Median (IQR)	Median (IQR)	F-value; P-value	P-value to Hedge’s g (95%CI)	P-value to Hedge’s g (95%CI)	P-value to Hedge’s g (95%CI)
6MWT (m)	443.5 (79)	388.5 (135)	336.5 (91)	F(2) = 30.6; P = 0.001*	P < 0.001** g = 0.6 (0.3 to 0.9)	P < 0.001** g = 0.88 (0.6 to 1.2)	P = 0.08 g = 0.25 (− 0.03 to 0.4)
TUG (s)	8.1 (2.8)	9.5 (2.6)	10.0 (2.6)	F(2) = 25.2, P = 0.001*	P < 0.001** g = − 0.5 (− 0.8 to − 0.2)	P < 0.001** g = − 0.6 (− 0.9 to − 0.3)	P = 0.4 g = − 0.1 (− 0.4 to 0.1)
QUEST device subscale (/5)	4.6 (0.9)	3.3 (0.8)	–	–	P < 0.001* g = 1.9 (1.0 to 2.8)	–	–
QUEST services subscale (/5)	5.0 (0.3)	4.6 (1.3)	–	–	P = 0.037 g = 0.6 (0.02 to 1.3)	–	–
QUEST total score (/5)	4.7 (0.7)	3.6 (0.8)	–	–	P < 0.001* g = 1.9 (1.0 to 2.7)	–	–
Peak dorsiflexion angle in early stance (°)	− 8.6 (4.9)	− 7.5 (5.9)	− 13.0 (8.0)	F(2) = 16.5; P < 0.001*	P < 0.001** g = − 0.7 (− 1.1 to − 0.3)	P = 0.055 g = − 0.5 (− 1.0 to − 0.02)	P < 0.001** g = − 1.0 (− 1.5 to − 0.4)
Peak dorsiflexion angle in swing (°)	− 4.2 (4.8)	− 4.0 (5.7)	− 16.9 (19.8)	F(2) = 13.7; P = 0.001*	P = 0.761 g = 0.007 (− 0.3 to 0.3)	P = 0.002** g = − 0.8 (− 1.3 to − 0.4)	P < 0.001** g = − 0.8 (− 1.2 to − 0.4)
Quasi-stiffness for the first linear part of the plantar/dorsiflexion moment–angle curve (dimensionless)	0.20 (0.10)	0.32 (0.20)	0.13 (0.08)	F(2) = 19.6; P = 0.001*	P = 0.007** g = − 0.7 (− 1.2 to − 0.2)	P = 0.008** g = − 0.4 (− 0.9 to − 0.03)	P < 0.001** g = − 1.7 (− 2.4 to − 1.0)
Peak plantarflexion angle in mid stance (°)	18.8 (6.8)	17.3 (5.3)	18.4 (5.6)	F(2) = 0.4; P = 0.819	–	–	–
Maximum dorsi/plantar flexion during swing (°)	1.1 (6.8)	0.0 (5.8)	− 3.5 (15.2)	F(2) = 4.13; P = 0.127	–	–	–
Peak plantarflexion moment in stance (dimensionless)	− 0.15 (0.03)	− 0.15 (0.04)	− 0.15 (0.04)	F(2) = 1.2; P = 0.549	–	–	–
Peak inversion moment in stance (dimensionless)	0.01 (0.01)	0.01 (0.01)	0.00 (0.01)	F(2) = 0.4; P = 0.819	–	–	–
Peak eversion moment in stance (dimensionless)	− 0.03 (0.04)	− 0.01 (0.03)	− 0.02 (0.03)	F(2) = 8.4; P = 0.015	–	–	–
Peak negative power in stance (dimensionless)	− 0.04 (0.01)	− 0.03 (0.02)	− 0.03 (0.01)	F(2) = 0.4; P = 0.819	–	–	–
Peak positive power in stance (dimensionless)	0.08 (0.03)	0.07 (0.04)	0.10 (0.05)	F(2) = 2.13; P = 0.344	–	–	–
Negative work in stance (dimensionless)	− 0.03 (0.01)	− 0.03 (0.01)	− 0.03 (0.01)	F(2) = 0.53; P = 0.766	–	–	–
Positive work in stance (dimensionless)	0.02 (0.01)	0.02 (0.01)	0.03 (0.01)	F(2) = 2.13; P = 0.344	–	–	–
Quasi-stiffness for the second linear part of the plantar/dorsiflexion moment–angle curve (dimensionless)	0.42 (0.30)	0.43 (0.19)	0.44 (0.20)	F(2) = 1.73; P = 0.420	–	–	–

Data are median (interquartile range)

*plsAFO* posterior leaf-spring AFO, *hAFO* helical AFO, *6MWT* 6-min walk test, *TUG* timed-up-and-go test, *P/F* plantar flexion

*Significant after Bonferroni correction if P-value < 0.0031. **Significant after Bonferroni correction if P-value < 0.017

Based on a difference of > 34 m between the noAFO [23] and each AFO condition, 3 participants were non responders with the hAFO and 12 were non-responders with the plsAFO. The median change in the plsAFO condition (25 m, IQR 59 m) did not reach the MCID, whereas the median change in the hAFO condition (74 m, IQR 72.3) was greater than the MCID.

### TUG

All 20 participants completed the TUG test. TUG performance time was significantly affected by the condition. The post-hoc analysis showed that this time was significantly shorter (i.e., better) with the hAFO than the plsAFO (Table 2). Data for each participant are shown in Fig. 3B.

Mean TUG performance time is 10.4 s (SD 2.3) in healthy individuals aged 60–64 years [40]. In the noAFO condition, 9 of the 20 participants were within this limit, this increased to 11 with the plsAFO and 16 with the hAFO. The MCID for the TUG test varies from 1 s [27] to 3.4 s [28]. The median change with the plsAFO (0.3 s, IQR 1.2) did not reach the MCID however the change improvement with the hAFO (1.6 s, IQR 1.8) was greater than some proposed MCID [27].

### 3D gait analysis

Complete kinematic and kinetic data with at least 4 gait cycles were available for 15 participants out of the 20. Technical issues occurred for 3 participants (e.g., they placed both feet on the same force platform preventing kinetic analysis). Only one cycle was available for 2 other participants which prevented appropriate averaging.

Three kinematic and kinetic variables were significantly affected by the condition and are presented below.

**Fig. 4:**
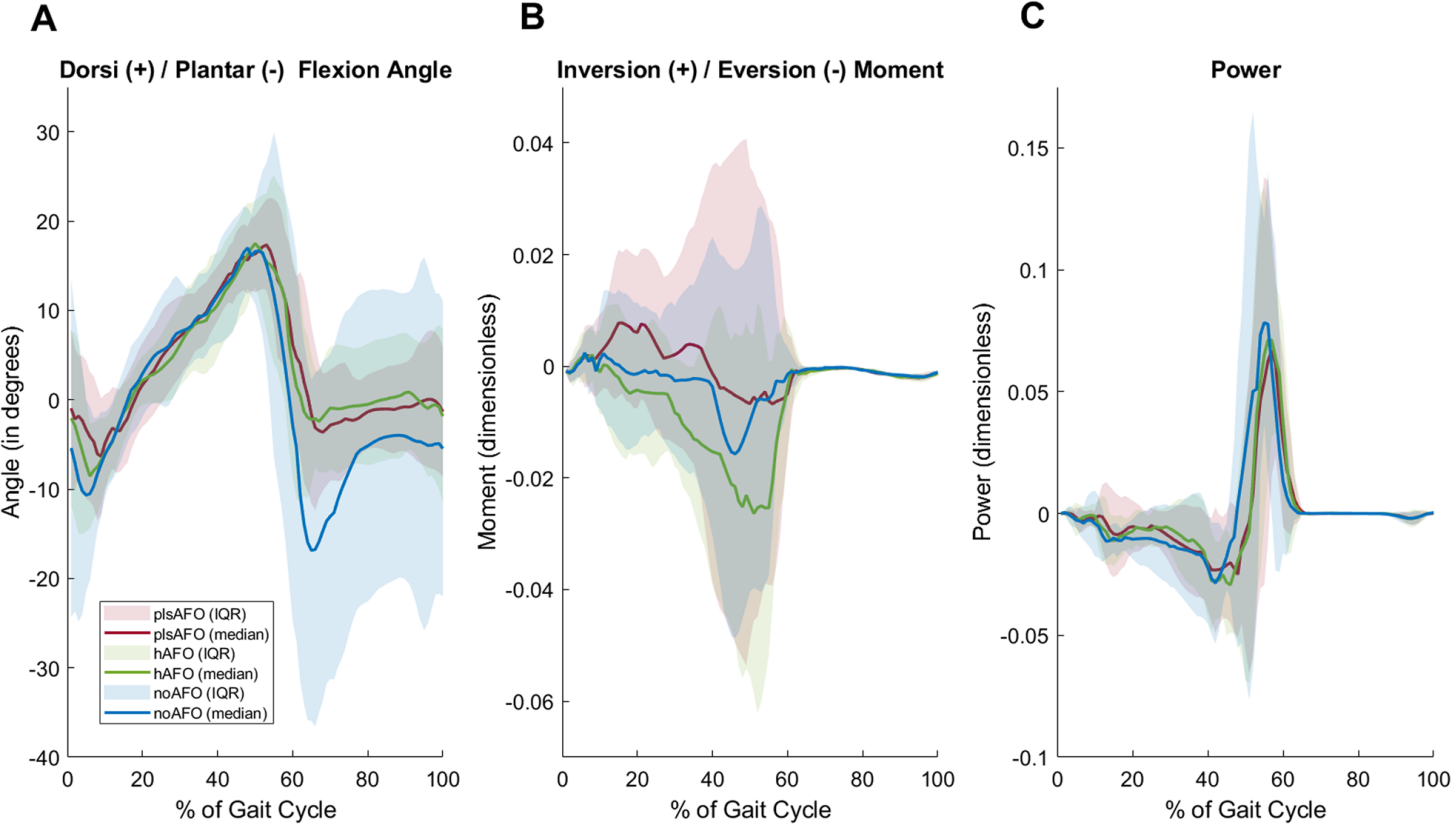
Median (IQR) curves for dorsiflexion/plantarflexion angle, eversion moment and power throughout the gait cycle for each condition (noAFO: shoes only, plsAFO: posterior ankle foot orthosis; hAFO: helical ankle foot orthosis)

First, peak plantarflexion during early stance was significantly affected by the condition. This peak was significantly lower with the plsAFO than the hAFO. It was also lower with the plsAFO than noAFO, with no difference between the hAFO and noAFO (Table 2). This result indicates that the plsAFO significantly limited plantarflexion during the loading response while the hAFO did not (Fig. 4A).

Second, peak plantarflexion during swing phase was significantly affected by the condition. This peak did not differ between the plsAFO and hAFO. However, it was lower with both AFOs than noAFO (Table 2). This result indicates that the plsAFO and hAFO prevented foot drop during swing (Fig. 4A).

**Fig. 5:**
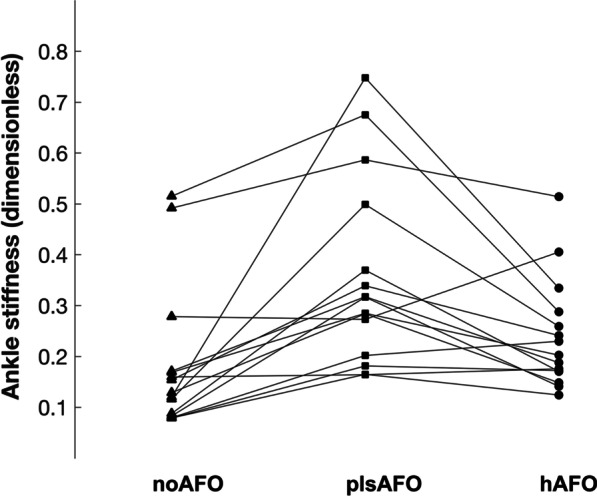
Results for each participant for non-dimensional quasi stiffness for the three conditions (noAFO: shoes only, plsAFO: posterior ankle foot orthosis; hAFO: helical ankle foot orthosis)

Third, dimensionless quasi-stiffness in the first linear part of the plantar/dorsiflexion moment–angle curve was significantly affected by the condition. This stiffness was significantly lower for hAFO than plsAFO. It was also significantly lower for noAFO than for plsAFO and hAFO (Table 2 and Fig. 5). This result indicates that quasi-stiffness was increased with both AFOs as compared with noAFO, but to a lesser extent with the hAFO.

No significant between-condition differences were found for any other kinematic and kinetic variables. However, the P-value for the effect of the condition on the dimensionless peak eversion moment in stance phase (which we considered to be representative of medio-lateral ankle stability) was quite low (P = 0.015) (Table 2). Furthermore, peak positive power was not affected by the condition (P = 0.34) (Fig. 4C), indicating that neither AFO reduced propulsion forces.

### The Quebec user evaluation of satisfaction with assistive technology (QUEST)

All participants completed the QUEST. Median QUEST scores for the device subscale were 3.2 (IQR 0.8) for the plsAFO and 4.6 (0.9) for the hAFO, corresponding to “more or less satisfied” and “quite satisfied-very satisfied” respectively. This score was significantly higher for the hAFO condition. Median scores for the services subscale were 4.6 (1.3) for the plsAFO and 5 (0.3) for the hAFO corresponding to “quite-very satisfied and “very satisfied” with no significant between group differences. Median total QUEST score was significantly higher for the hAFO: 4.7 (0.7) versus 3.6 (0.8). The results for each item are shown in Fig. 3C.

### Harms

No harms were reported by any participants at any assessment for either type of AFO.

## Discussion

To our knowledge, this is the first study of a helical-shaped ankle–foot orthosis (hAFO) designed to reduce foot drop and improve medial–lateral stability of the ankle in individuals with peripheral neuropathy or NMD, with no spasticity. The results showed that the helical orthosis increased functional gait distance (6MWT) and functional mobility (TUG test performance time) statistically significantly and considerably more than the posterior leaf spring orthosis. Furthermore, the kinematic and kinetic analyses showed that the hAFO allowed more physiological ankle mobility whilst providing some lateral stability, which was not the case for the plsAFO. Participants’ rating of satisfaction was significantly higher with the hAFO than the plsAFO.

The majority of studies performed to evaluate AFOs have been conducted in individuals with stroke or cerebral palsy, and a much smaller number have been conducted in people with peripheral neurological or neuromuscular conditions [10, 41]. The requirements of these conditions differ, for example, people with central neurological disorders and spastic hypertonia often require more rigid orthoses to cope with the more widespread and severe impairments that result from stroke [7]. In contrast, the participants in the present study had no spastic hypertonia and more focal weakness. Their requirement was for an orthosis that prevented excessive plantarflexion and/or inversion during the swing phase of gait while allowing normal mobility to optimize function.

### Impact of the helical orthosis on functional gait distance and mobility

6MWT distance was significantly greater with the hAFO than the plsAFO, with a moderate effect size. Furthermore, almost all participants could be considered as ‘responders’ to the hAFO, which was not the case for the plsAFO, and the median change from noAFO with the hAFO largely surpassed the MCID, whereas change with the plsAFO did not reach the MCID.

Comparison with the literature found smaller improvements in 6MWT distance with different types of AFO, although few studies included samples similar to our sample. In people with stroke, the use of a posterior AFO improved 6MWT distance by approximately 30 m compared with no AFO [20, 42]. Other results in the literature regarding the effect of orthoses on gait speed in individuals with neuromuscular conditions are variable. One study, which compared three off-the-shelf, commercially available AFOs in individuals with Charcot-Marie-Tooth disease, found no improvements in gait speed or any spatiotemporal variables compared with a shoe-alone condition, despite significant improvements in dorsiflexion angle during swing [4]. Another study in individuals with FSH found improvements in gait speed with custom foot orthoses (for those with less severe impairment) and with custom rigid carbon fibre orthoses (for those with more severe impairment) [41]. In contrast, another study found that an off-the-shelf posterior leaf spring orthosis actually reduced gait speed [10]. The increase in 6MWT distance with the hAFO in the present study appears to be substantially greater than the improvements in distance or speed found in the above-mentioned studies.

TUG test performance time was significantly shorter (i.e., better) with the hAFO than the plsAFO. The TUG test measures several aspects of gait including sit-stand capacity, speed and balance. The better TUG performance with the hAFO likely results from a combination of a higher gait speed because of the reduction in foot drop, and better stability, as indicated by the increased eversion moment for some participants, which may have facilitated balance and increased turning speed. Studies in subacute or chronic stroke found greater improvements in TUG performance time than we did in the present study (up to a mean 6 s difference between various types of AFO and noAFO conditions) [43]. This discrepancy can be explained by the fact stroke is very different to the pathologies of the participants here, and the fact that the median (IQR) TUG performance time in the noAFO condition was 10.0 (2.6) which is close to the 10.4 s (SD 2.3) reported by in healthy 60–64 year old individuals [40], therefore a ceiling effect may have reduced the potential for change. We were unable to find studies that evaluated pathologies similar to those in the present study.

### Effect of the helical orthosis on biomechanical gait variables

This is one of the first studies to evaluate the impact of AFOs on biomechanical gait variables in patients with peripheral neuropathy or NMD. The purpose of this analysis was to explain the results of the functional gait tests, however fewer significant effects of the condition were found on kinematic and kinetic variables than expected. This could result from several factors. First, we controlled the gait velocity: participants were constrained to walk at the same speed as the noAFO condition in both AFO conditions. Gait speed was therefore sub-maximal in the AFO conditions since participants were able to walk faster with the AFOs, as demonstrated by the 6MWT results. We chose this design to differentiate the specific effects of the AFO on ankle biomechanics from the more general effect, which is typically an increase in gait velocity. Second, as can be seen in Table 1, mean plantarflexion strength was rated at 4 on the MRC scale, indicating that most participants had functional strength in these muscles. This could explain the lack of impact of the orthoses on variables such as work and power. The analyses may also have been underpowered since this analysis did not include data from the whole sample (n = 15). It was interesting, however, that the 3 significant effects found corresponded directly to some design features of the AFO.

The primary aim of an AFO is to prevent foot drop during swing phase. Median peak plantarflexion during swing was above − 5 deg with both the plsAFO and hAFO, showing that the support provided under the forefoot reduced plantarflexion. Our next aim in the design of the hAFO was that it should not interfere with ankle mobility. The results showed that this was achieved with the hAFO whereas the plsAFO limited plantarflexion during the loading response. However, although the median difference in this plantarflexion angle was significant between the hAFO and the plsAFO, it was only of 1.1° and therefore could result from measurement error. Furthermore, although quasi-stiffness during stance was higher with the hAFO than in the noAFO condition, the value was lower than with the plsAFO. Quasi-stiffness provides an indication of overall joint stiffness, including muscles and ligaments combined with the stiffness of the AFO [34]. Although it is not possible to separate the contribution of the AFO from the contribution of active and passive anatomical structures from the AFO [34] the results suggest that the helical design of the hAFO created less resistance to plantarflexion than the plsAFO.

The hAFO was designed with a heel cup to provide ankle stability in the frontal plane since people with peripheral neuropathy or NMD often have weakness of the evertor muscles. The inversion/eversion moment has rarely been studied in the literature in comparison with the plantar/dorsiflexion moment [7]. The analysis revealed that the hAFO generated a higher (though not significantly) peak eversion moment than the plsAFO or noAFO, suggesting that this orthosis provided some lateral stability in the stance phase of gait. The lack of significance and small effect size might be due to the heterogeneity of evertor weakness in the sample: 5 participants had a score of 4 on the MRC scale for evertor muscle strength. However, the stabilising effect on participants with instability may have improved their confidence and is likely related to the large increase in 6MWT distance and decrease in TUG performance time.

Although the hAFO was also designed to increase propulsion thanks to its spring like behaviour (carbon material and helical shape), we did not demonstrate this effect. However, this could not be fully analysed due to the design of the study which involved a controlled gait speed. However, in contrast with some other orthoses [4] the hAFO at least did not reduce peak positive power. Future studies should investigate the impact of the hAFO on propulsion at spontaneous gait speeds.

### Satisfaction

Few studies have assessed patient satisfaction with AFOs using a validated questionnaire. We used the QUEST for this purpose since it has good reliability and validity [44]. Evaluation of satisfaction is an essential component of the validation process since if the end-users are not sufficiently satisfied with a produce, they will simply not use it, unless their gait impairment is sufficiently severe [12]. The results of the QUEST showed that participants were significantly more satisfied with all the device related aspects of the hAFO compared with the plsAFO, including weight, safety, comfort and effectiveness. There were no differences between the AFOs in terms of service scores, however this is not surprising since the services were provided by the same company.

A study of carbon fibre Toe-OFF and BlueRocker AFOs found that 109 of 123 patients with neuromuscular disease preferred these orthoses to their previous AFOs because they found them lighter, cooler, they provided push-off and allowed them to wear a normal sized shoe [45], thus demonstrating the importance of comfort and aesthetics in the design of AFOs. This was confirmed in a recent, large survey of individuals with Charcot-Marie-Tooth disease that found that appearance, discomfort, abrasions and pain could lead to dissatisfaction with an orthosis [13]. The QUEST did not specifically evaluate all these dimensions; however, comfort was highly rated with the hAFO, and the items for dimensions and weight (which could relate to appearance) were rated quite-very satisfied, suggesting that the hAFO fulfilled these criteria. Indeed, hAFOs weigh around 250 g whereas plsAFOs are 50–100 g heavier.

### Limitations

The inclusion of a heterogenous sample of participants with peripheral neurological and neuromuscular disorders could be considered as a limitation. However, all participants were able to walk for at least 6 min with noAFO (but gait aids such as sticks were allowed), which is typical of AFO users with foot drop that is not of central neurological origin. The helical orthosis evaluated is thus suitable for this population.

No recommendations exist regarding the time required for individuals to accommodate to a new AFO and adapt their gait. Therefore, we do not know if 7 days was sufficient for accommodation to have fully occurred.

We were unable to fully analyse the effect of the AFOs on propulsion owing to the sub-maximal gait speed imposed on participants. Further studies should therefore evaluate the biomechanical effects of the hAFO at the participants’ own, spontaneous gait speed.

The fact all the orthoses were made by the same orthotist ensured homogeneity in the moulding and manufacturing of the orthoses; however, future studies should evaluate the repeatability of hAFOs made by different orthotists.

## Conclusions

This study showed promising improvements in some outcome measures for a small group of individuals with peripheral neuropathy or NMD who have drop foot. The new hAFO improves gait capacity more, may have better biomechanical function, is more aesthetic, is lighter in weight and induces higher levels of satisfaction than the plsAFO. These results suggest that the hAFO can be prescribed for individuals with peripheral neuropathy or neuromuscular disease who have drop foot and/or ankle instability that reduces their gait capacity.

## Data Availability

The datasets used and/or analysed during the current study are available from the corresponding author on reasonable request.

## Abbreviations

AFO: Ankle–foot orthosis
CI: Confidence interval
FSH: Facioscapulohumeral muscular dystrophy
hAFO: Helical ankle foot orthosis
IQR: Interquartile range
MCID: Minimum clinically important difference
MRC scale: Medical Research Council scale
NMD: Neuromuscular disease
plsAFO: Posterior leaf spring ankle foot orthosis
QUEST: Quebec user evaluation of satisfaction with assistive technology
SD: Standard deviation
TUG test: Timed up and go test
6MWT: 6-Minute walk test
